# Supplementary material for: Improving variant calling using population data and deep learning
Source: BMC Bioinformatics. 2023 May 12;24:197. doi: 10.1186/s12859-023-05294-0 (PMC10182612; doi:10.1186/s12859-023-05294-0)
Supplement: Supplementary file 1 — Additional file 1. Supplementary Information. [file 12859_2023_5294_MOESM1_ESM.pdf]

Supplementary Information for: Improving variant calling using population data and deep learning

Nae-Chyun Chen[1, ‡, *], Alexey Kolesnikov[2], Sidharth Goel[2], Taedong Yun[2], Pi-Chuan Chang[2, †], and Andrew Carroll[2, †, *]

[1]Department of Computer Science, Johns Hopkins University, Baltimore, MD 21218, USA
[2]Google Health, Palo Alto, CA 94304 and Cambridge, MA 02142, USA
corresponding author: cnaechy1@jhu.edu; awcarroll@google.com
[†]These authors contributed equally to this work.
[‡]Work performed while an intern at Google Health.

March 30, 2023

Supplementary Notes

S1 Performance on zero-frequency variants

A potential concern for population-aware variant calling models is increasing false negative rate for novel alleles. Since it is not trivial to define a set of truly novel variants in the 1000 Genomes Project, we extracted variants with zero allele frequency to investigate the impact when population information is included in a variant calling model. Using the GIAB v4.2.1 truth set, there are 32,256 (1.0%) SNPs and 3,193 (0.6%) indels that have zero allele frequency for sample HG003. We then use the zero-frequency variant set to evaluate recall of actual variant calls using hap.py[1].

We observed that the recall on zero-frequency variants underperforms the rest using all DeepVariant models, regardless of variant types and whether to utilize population information (Figure S1). With 35x reads, the recall of the population-agnostic DeepVariant model (DeepVariant) is 0.8938 for SNPs and 0.7337 for indels. The recall further decreases to 0.8813 for SNPs and 0.7142 for indels when using DeepVariant-AF. When using 21x reads, the drop in accuracy gets larger for both types of variants. This is consistent with our analysis that the population-aware DeepVariant model requires stronger evidence

(higher-quality pileup images) to call zero-frequency variants, thus reducing recall. Further, the population information has a stronger influence in variant calling for low-coverage datasets. Despite the disadvantages, the negative impact on zero-frequency variants is small compared to overall error reduction.

To better understand the zero-frequency variants, we called variants using the DeepVariant PacBio model with the PrecisionFDA v2 35x HG003 reads set sequenced with the PacBio HiFi technology[2]. The recall for the zero-frequency variants improves to 0.9519 for SNPs and 0.9132 for indels. The large difference in recall/FNR indicates that many of the zero-frequency variants are hard to genotype using Illumina reads, and may not be novel mutations relative to samples in reference panels. In the future, reference panels utilizing high-quality long reads will likely provide better allele frequency estimates and improve the population-aware model performance[3–5].

Supplementary Tables

Table S1: Variant calling results for WGS HG003.

Coverage	Type	Caller	Precision	Recall	F1
35x	INDEL	DeepVariant-AF	0.994056	0.997466	0.995758
		DeepVariant	0.993992	0.997365	0.995676
		GATK	0.991594	0.991121	0.991357
		Octopus	0.989744	0.996324	0.993023
		Strelka2	0.987627	0.995968	0.99178
	SNP	DeepVariant-AF	0.993929	0.998499	0.996208
		DeepVariant	0.993824	0.998177	0.995996
		GATK	0.992233	0.9884	0.990313
		Octopus	0.988984	0.995357	0.99216
		Strelka2	0.992187	0.981123	0.986624
21x	INDEL	DeepVariant-AF	0.984279	0.992219	0.988233
		DeepVariant	0.982706	0.991749	0.987206
		GATK	0.975544	0.982909	0.979213
		Octopus	0.97672	0.99065	0.983636
		Strelka2	0.958601	0.989087	0.973605
	SNP	DeepVariant-AF	0.991864	0.996686	0.994269
		DeepVariant	0.991698	0.996023	0.993856
		GATK	0.98848	0.987216	0.987847
		Octopus	0.984764	0.993798	0.989261
		Strelka2	0.981011	0.992999	0.986968
10x	INDEL	DeepVariant-AF	0.889863	0.958186	0.922762
		DeepVariant	0.880882	0.953219	0.915624
		GATK	0.865516	0.944581	0.903322
		Octopus	0.889788	0.9577	0.922496
		Strelka2	0.750313	0.96193	0.843045
	SNP	DeepVariant-AF	0.952551	0.985066	0.968536
		DeepVariant	0.950283	0.978971	0.964414
		GATK	0.939326	0.976279	0.957446
		Octopus	0.938012	0.984203	0.960552
		Strelka2	0.916328	0.976271	0.94535
6x	INDEL	DeepVariant-AF	0.72068	0.914249	0.806005
		DeepVariant	0.710767	0.902276	0.795153
		GATK	0.691391	0.898816	0.781576
		Octopus	0.748593	0.905851	0.819748
		Strelka2	0.500348	0.935336	0.651945
	SNP	DeepVariant-AF	0.82965	0.962828	0.891292
		DeepVariant	0.82624	0.948907	0.883335
		GATK	0.80301	0.951194	0.870843
		Octopus	0.834285	0.953795	0.890047
		Strelka2	0.753403	0.917202	0.827272

Table S2: Variant calling results for WES HG003 (Oslo dataset)

Type	Caller	False negatives	False positives	Recall	Precision	F1 score
INDEL	DeepVariant-AF	56	30	0.965389	0.981343	0.973301
	DeepVariant	61	35	0.962299	0.978234	0.970201
	GATK	113	182	0.930161	0.893255	0.911334
	Octopus	82	71	0.94932	0.956996	0.953143
	Strelka2	104	80	0.935723	0.950403	0.943006
SNP	DeepVariant-AF	270	100	0.990198	0.996347	0.993263
	DeepVariant	279	112	0.989871	0.995909	0.992881
	GATK	283	365	0.989726	0.986784	0.988252
	Octopus	456	213	0.983445	0.99219	0.987798
	Strelka2	379	138	0.98624	0.994945	0.990574

Table S3: Variant calling results for WES HG003 (IDT dataset)

Type	Caller	False negatives	False positives	Recall	Precision	F1 score
INDEL	DeepVariant-AF	28	14	0.975779	0.987952	0.981827
	DeepVariant	31	21	0.973183	0.981974	0.977559
	GATK	74	315	0.935986	0.780181	0.851011
	Octopus	52	81	0.955017	0.933168	0.943966
	Strelka2	63	143	0.945502	0.886328	0.914959
SNP	DeepVariant-AF	258	131	0.989845	0.994818	0.992326
	DeepVariant	282	135	0.988901	0.994656	0.99177
	GATK	266	433	0.98953	0.98306	0.986285
	Octopus	437	216	0.9828	0.991418	0.98709
	Strelka2	365	141	0.985634	0.994401	0.98999

Table S4: Recall for HG003 GIAB v4.2.1 variants that have zero allele frequency in the 1000 Genomes Project. Values in the parentheses in the "Type" column specify the total number of zero-allele-frequency variants for each variant type

Type	DeepVariant-AF	DeepVariant	GATK	Octopus	Strelka2
INDEL (1,592)	0.714196	0.733668	0.837312	0.789573	0.739322
SNP (1,365)	0.881319	0.893773	0.898168	0.882051	0.890842

Table S5: Variant calling accuracy on common (allele frequency > 0.01) variants. Dataset: HG003 35x WGS

**Type**	**Caller**	**False negatives**	**False positives**	**Recall**	**Precision**	**F1 score**
INDEL	DeepVariant-AF	2055	1361	0.995829	0.997351	0.9965894
	DeepVariant	2179	1345	0.995577	0.997382	0.9964787
	GATK	3465	2565	0.992967	0.994999	0.9939820
	Strelka2	5344	2104	0.989153	0.995855	0.9924927
SNP	DeepVariant-AF	10209	4401	0.996835	0.998634	0.9977337
	DeepVariant	10979	4415	0.996597	0.998629	0.9976120
	GATK	16202	13630	0.994978	0.995773	0.9953753
	Strelka2	29972	3925	0.990709	0.998774	0.9947252

Table S6: Variant calling accuracy on rare (allele frequency ≤ 0.01) variants. Dataset: HG003 35x WGS

**Type**	**Caller**	**False negatives**	**False positives**	**Recall**	**Precision**	**F1 score**
INDEL	DeepVariant-AF	949	207	0.919583	0.983799	0.9506077
	DeepVariant	866	306	0.926616	0.976500	0.9509042
	GATK	635	2221	0.946191	0.859350	0.9006821
	Strelka2	998	1059	0.915431	0.925794	0.9205833
SNP	DeepVariant-AF	4033	678	0.943084	0.992656	0.9672353
	DeepVariant	3876	1750	0.945300	0.981345	0.9629853
	GATK	3980	25226	0.943832	0.784869	0.8570418
	Strelka2	4338	3079	0.938780	0.967704	0.9530226

Table S7: Software used in the experiments

DeepVariant[6]	1.1
GATK[7]	4.2.0.0
Octopus[8]	0.7.2
Strelka2[9]	2.9.2
GNU Parallel[10]	20200322

Supplementary Figures

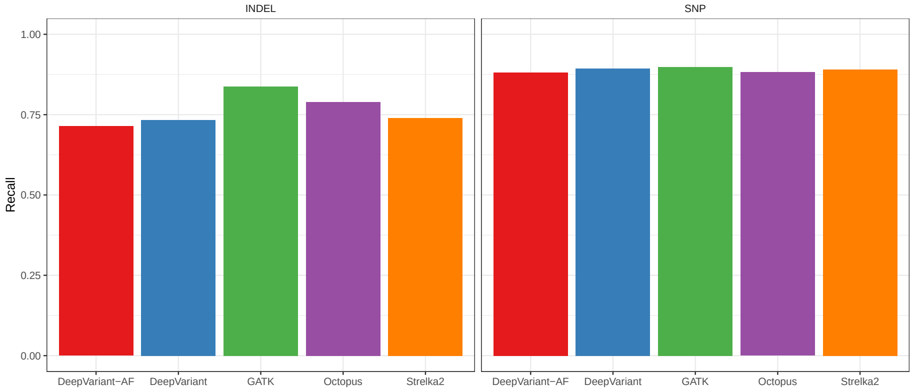

Figure S1: Recall for HG003 GIAB v4.2.1 variants that have zero allele frequency in the 1000 Genomes Project

References

1. Krusche, P., Trigg, L., Boutros, P. C., Mason, C. E., Francisco, M., Moore, B. L., Gonzalez-Porta, M., Eberle, M. A., Tezak, Z., Lababidi, S., *et al.* Best practices for benchmarking germline small-variant calls in human genomes. *Nature biotechnology* **37,** 555–560 (2019).
2. Olson, N. D., Wagner, J., McDaniel, J., Stephens, S. H., Westreich, S. T., Prasanna, A. G., Johanson, E., Boja, E., Maier, E. J., Serang, O., *et al.* precisionFDA Truth Challenge V2: Calling variants from short-and long-reads in difficult-to-map regions. *bioRxiv* (2020).
3. Ebert, P., Audano, P. A., Zhu, Q., Rodriguez-Martin, B., Porubsky, D., Bonder, M. J., Sulovari, A., Ebler, J., Zhou, W., Mari, R. S., *et al.* Haplotype-resolved diverse human genomes and integrated analysis of structural variation. *Science* **372** (2021).
4. De Coster, W., Weissensteiner, M. H. & Sedlazeck, F. J. Towards population-scale long-read sequencing. *Nature Reviews Genetics,* 1–16 (2021).
5. Beyter, D., Ingimundardottir, H., Oddsson, A., Eggertsson, H. P., Bjornsson, E., Jonsson, H., Atlason, B. A., Kristmundsdottir, S., Mehringer, S., Hardarson, M. T., *et al.* Long-read sequencing of 3,622 Icelanders provides insight into the role of structural variants in human diseases and other traits. *Nature Genetics* **53,** 779–786 (2021).
6. Poplin, R., Chang, P.-C., Alexander, D., Schwartz, S., Colthurst, T., Ku, A., Newburger, D., Dijamco, J., Nguyen, N., Afshar, P. T., *et al.* A universal SNP and small-indel variant caller using deep neural networks. *Nature biotechnology* **36,** 983–987 (2018).
7. Van der Auwera, G. A. & O'Connor, B. D. *Genomics in the Cloud: Using Docker, GATK, and WDL in Terra* (O'Reilly Media, 2020).
8. Cooke, D. P., Wedge, D. C. & Lunter, G. A unified haplotype-based method for accurate and comprehensive variant calling. *Nature biotechnology,* 1–8 (2021).
9. Kim, S., Scheffler, K., Halpern, A. L., Bekritsky, M. A., Noh, E., Källberg, M., Chen, X., Kim, Y., Beyter, D., Krusche, P., *et al.* Strelka2: fast and accurate calling of germline and somatic variants. *Nature methods* **15,** 591–594 (2018).
10. Tange, O. *GNU Parallel 2018* ISBN: 9781387509881. `https://doi.org/10.5281/zenodo.1146014` (Ole Tange, Mar. 2018).
